# Predicting Outcome after Total Hip Arthroplasty: The Role of Preoperative Patient-Reported Measures

**DOI:** 10.1155/2019/4909561

**Published:** 2019-01-29

**Authors:** Markus Weber, Florian Zeman, Benjamin Craiovan, Max Thieme, Moritz Kaiser, Michael Woerner, Joachim Grifka, Tobias Renkawitz

**Affiliations:** ^1^Department of Orthopaedic Surgery, Regensburg University Medical Center, Bad Abbach, Germany; ^2^Center of Clinical Studies, Regensburg University Medical Center, Germany

## Abstract

Choosing the appropriate patient for surgery is crucial for good outcome in total hip arthroplasty (THA). Therefore, parameters predicting outcome preoperatively are of major interest. In the current study, we compared the predictive power of different presurgical measures in minimally invasive THA. In the course of a prospective clinical trial preoperative HOOS, EQ-5D and SF-36 were obtained in 140 patients undergoing THA. Responder rate was defined by the modified OMERACT-OARSI criteria at six-month-, one-year, two-year, and three-year follow-up. Logistic regression was performed to compare the different questionnaires regarding their power of predicting positive responders. ROC-curve analysis was used to define benchmarks in preoperative measures associated with good outcome. Preoperative HOOS (p<0.001), EQ-5D (p=0.007), and PCS of SF-36 (p<0.001) were higher in responders than in nonresponders whereas no differences between responders and nonresponders were found for preoperative MCS (p=0.96) of SF-36. However, preoperative HOOS revealed best predictive power (OR=0.84 95%CI=0.78-0.90, p<0.001, Pseudo R-Squared according to Nagelkerke=0.48, effect size according to Cohen=0.96) compared to all other preoperative measures. Multivariable analysis confirmed preoperative HOOS as an independent parameter correlating with postoperative responder status (OR=0.76, 95% CI=0.66–0.88, p<0.001). In ROC-curve analysis nonresponders were identified with a sensitivity of 91.7% and specificity of 68.9% using a cutoff in preoperative HOOS of 40.3. Presurgical HOOS can predict outcome in THA better than other preoperative outcome measures. Patients with a preoperative HOOS value less than 40.3 have the highest probability of a positive response in terms of pain and function after THA.

## 1. Introduction

Total hip arthroplasty (THA) is one of the most frequently performed procedures in orthopaedic surgery [1]. It represents a curative treatment option of advanced hip arthritis with the capacity to substantially improve quality of life [2]. For primary THA an increase of 174% is estimated in the United States by 2030 [3]. The high demand for THA is faced with restricted clinical resources, thus leading to prolonged wait times and even a potential supply side crisis [4, 5]. Despite all technical advances in THA over the last decades, there is still a certain number of dissatisfied patients with residual pain and function deficits regarding the postoperative outcome after THA [6]. Therefore, it is of great interest in orthopaedic surgery to identify predictors for good outcome. This might facilitate handling the indication of THA with high responsibility and choosing the appropriate patients for surgery especially in times of limited resources. Patient-reported preoperative measures (PROMs) have routinely been introduced by the national health systems and quality networks to ensure clinical standards and supervise outcome after THA [7, 8]. Originally PROMs were designed for clinical studies and were shown to measure outcomes after THA with high validity and reliability [9, 10]. Although PROMs were not developed to predict outcomes, different studies showed an association between presurgical values of these questionnaires and postoperative outcomes [2, 11–13]. However, the discussion in literature is controversial [14, 15]. In the current single center study we evaluated three of the most commonly used presurgical measures in THA regarding their ability to predict postoperative outcome in THA. We asked which preoperative measure shows the best correlation to positive responders after THA at a high volume center for arthroplasties. Second, we tried to define preoperative benchmark values for the best preoperative measure to identify good responders in THA with a high sensitivity.

## 2. Patients and Methods

In the course of a prospective clinical trial, 140 patients underwent minimally invasive THA. The investigation was registered in the Clinical Trial Register (DRKS00000739, German Clinical Trials Register) and approved by the local ethics commission. According to the protocol of the main study [16, 17], eligible participants were patients between the ages of 50 and 75 years with an American Society of Anaesthesiologists (ASA) score of 3 or below who were admitted for primary cementless unilateral THA attributable to primary or secondary osteoarthritis. Exclusion criteria were age younger than 50 years (as a postoperative CT scan was required) and older than 75 years (to ensure postoperative follow-up was achieved), ASA score greater than 3, arthritis attributable to hip dysplasia, posttraumatic hip deformities, and previous hip surgery. Only patients who had no significant disease of the contralateral hip were included. Because of the strict inclusion criteria, of 783 patients screened, 597 did not meet the inclusion criteria. Twenty-seven patients declined to participate and 19 were excluded for other reasons (e.g., cancellation of the operation owing to elevated inflammatory markers).

**Figure 1:**
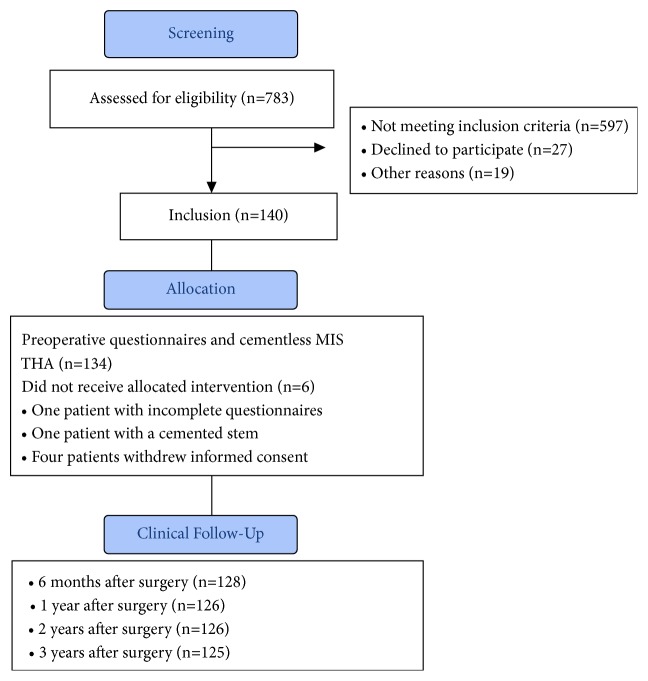
Flow diagram of the study participants.

Prior to participation in the study a written informed consent was obtained. THA was performed with all patients in the lateral decubitus position using a minimally invasive single-incision anterolateral approach by four experienced orthopaedic surgeons, three of which are among the coauthors (MWo, JG, TR) in our Department of Orthopaedic Surgery, Regensburg University Medical Center, Bad Abbach, Germany. Press-fit acetabular components and cement-free hydroxyapatite-coated stems (Pinnacle®cup, Corail®stem; DePuy, Warsaw, IN, USA) with metal heads of 32 mm were used. Of the initially 140 patients, four withdrew their informed consent and thus had to be excluded. For one further patient, preoperative questionnaires were incomplete, and for one patient a cemented stem had to be used due to severe osteoporosis, leaving 134 patients. For six-month follow-up 128, for one-year 126, for two-year 126, and for 3-year 125 patients were available to define responder status (Figure 1). Anthropometric characteristics of the study group are shown in Table 1.

Preoperatively measures such as the Hip Disability and Osteoarthritis Outcome Score (HOOS) [18], EuroQol (EQ-5D) [19], and the Short Form 36 questionnaire (SF-36) [20] were obtained. These scores are usually obtained to measure outcome after THA. In this study we only used the preoperative values of these scores to investigate which score is best at predicting patients with good outcome after THA. The HOOS was developed to measure outcome in patients with hip osteoarthritis. All Western Ontario and McMaster Universities Osteoarthritis Index (WOMAC) [21] questions are included in the HOOS. In addition to WOMAC, HOOS contains subscales for sport and recreation function resulting in a better responsiveness especially in younger patients [22]. The HOOS consists of 5 subscales: pain, symptoms, activities of daily living, sport, and quality of life built by 40 items. For standardized answers five Likert-boxes are available. The best scale is 100 points indicating no problems [9]. The EQ-5D is a widely used and tested descriptive instrument for evaluating health. It defines health based on five dimensions: Mobility, Self-Care, Usual Activities, Pain/Discomfort, and Anxiety/Depression. Each dimension has 3 response categories ranging from no problems and some problems to extreme problems. The EQ-5D was tested in general population and patient samples for valuing health [23]. The SF-36 is a common general health scale evaluating physical and mental health. It measures three major health attributes such as functional status, well-being, and overall health in eight subscales. These consist of physical function, pain, health, vitality, social function, emotional health, and mental health. The responses of the 36 questions are transferred to 0-100 worst/best scale whereas 50 points correspond to a general healthy population [20, 24]. To generate summary scores country specific weights were generated. As a result, the Physical Component Summary (PCS) and the Mental Component Summary (MCS) can be built [25].

For dichotomizing responders and nonresponders at each follow-up point after THA, the Outcome Measures in Rheumatology and Osteoarthritis Research Society International (OMERACT-OARSI) consensus responder criteria were used [26, 27]. Responder status was defined separately for each follow-up point. The OMERACT-OARSI criteria assess responder status based on relative change in WOMAC scores in relation to benchmarks determined by expert consensus and statistical analyses. The WOMAC itself is an international widely used score to evaluate outcome after total joint replacement representing a multidimensional measure of pain, stiffness, and physical functional disability [28]. This measurement of outcome has especially been developed for patients with osteoarthritis and has been approved in several longitudinal studies with patients undergoing total joint replacement [29–31]. For defining responders we chose the OMERACT-OARSI criteria since they do not depend on patient characteristics of the cohort, thus reducing any potential selection bias [32]. The OMERACT-OARSI criteria to assess responders after total joint replacement were previously described [2, 33]. Due to the low numbers of true nonresponders, we set the OMERACT-OARSI criteria stricter to define patients with high response after THA. The modified criteria defined a patient as a responder if all single requirements of the OMERACT-OARSI criteria [26] were met at the same time. This comprised improvement in pain and function of at least 50% and absolute change of at least 20 points. Therefore, our modified criteria implicate high improvement in both pain and function postoperatively.

For statistical analysis, continuous data are presented as median (range) due to nonnormal distribution of presurgical measures. Accordingly, group comparisons were performed using Mann-Whitney U-tests. Absolute and relative frequencies were given for categorical data and compared between groups by chi-square tests. Logistic regression was performed for preoperative measures with significant differences between responders and nonresponders (HOOS, EQ-5D, PCS) to evaluate sensitivity in predicting responders at each follow-up point after surgery. To allow a direct comparison between the different scores in the regression models the EQ-5D index value was transformed to a 0-100 scale by multiplying by 100. Pseudo R-Squared according to Nagelkerke and Cohen effect size were calculated to evaluate the quality of each model. A Cohen value of 0.01 represents a weak effect, a value of 0.25 a middle effect, and a value of 0.4 a strong effect, respectively [34]. Furthermore, odds ratios (OR) were compared between the preoperatively obtained measures. Afterwards a multivariable logistic regression model including age, gender, ASA, BMI, operative time, Kellgren Score ranging from 0 to 10 points, (grade 0 = 0 points, grade 1= 1-2 points, grade 2 = 3-4 points, grade 3 = 5-9 points, grade 4 = 10 points), length of skin incision, preoperative pain level, and preoperative expectations was calculated to test the independent correlation of the best score with one-year responder rate. Then receiver operating characteristic (ROC) curve plots were generated for each follow-up point (6 months, 1 year, 2 years, 3 years). The Youden Index was used to define benchmarks to predict outcome after THA with the help of preoperative measures. The Youden indices, sensitivity, specificity, and area under the curve (AUC) were compared between the different follow-up points. IBM SPSS Statistics 22 (SPSS Inc., Chicago, IL, USA) was used for analysis.

## 3. Results

**Figure 2:**
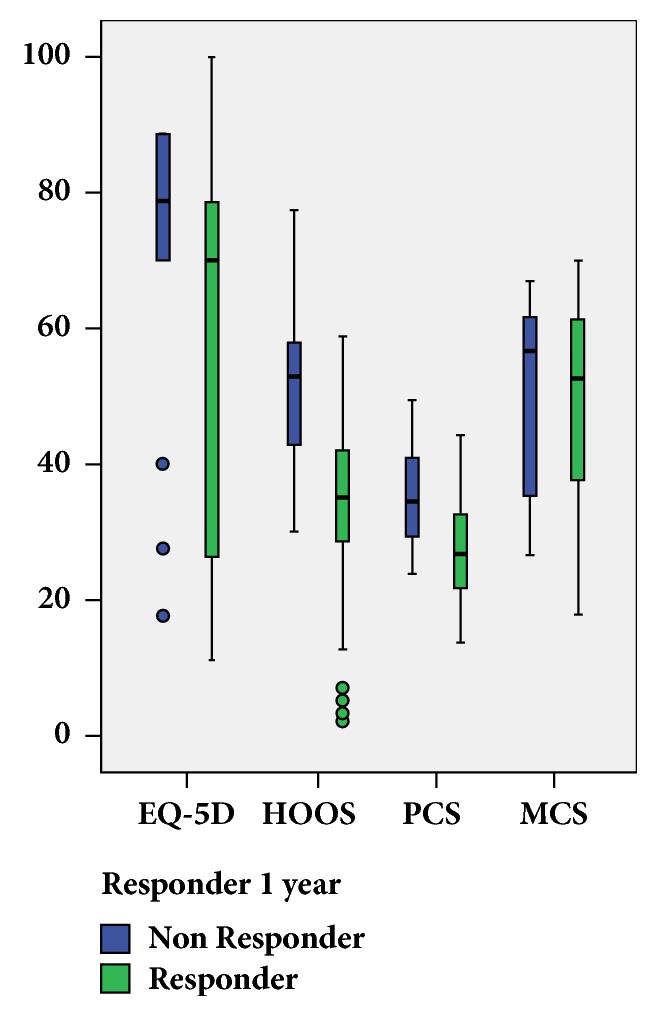
Preoperative measures (HOOS, EQ-5D, PCS, MCS) for responders and nonresponders assessed one year after total hip arthroplasty. EQ-5D°: EuroQol multiplied by 100, HOOS: Hip Disability and Osteoarthritis Outcome Score, PCS: Physical Component Summary of the Short Form 36 questionnaire, MCS: Mental Component Summary of the Short Form 36 questionnaire.

Using the stricter modified OMERACT-OARSI criteria [26] one year after THA, we found 103 responders and 23 nonresponders. Preoperative HOOS (p<0.001), EQ-5D (p=0.007) and PCS of SF-36 (p<0.001) were higher in responders than in nonresponders whereas no differences between responders and nonresponders were found for preoperative MCS (p=0.96) of SF-36 (Figure 2). For the preoperative HOOS, EQ-5D, and PCS, this held also true for 6 month-, two-year, and three-year responders (Table 2).

Since HOOS, EQ-5D, and PCS showed significant differences between responders and nonresponders, binary regression analyses were performed for these questionnaires. Analyzing the relation to responder grade one year after THA, preoperative HOOS revealed best Pseudo R-Squared according to Nagelkerke with 0.48 and a corresponding effect size according to Cohen with 0.96. Similarly, OR showed the strongest correlation between responder status one year after surgery and preoperative HOOS with 0.84 (95% CI = 0.78 - 0.90, p<0.001) compared to other presurgical measures. This held true for all follow-up points (Table 3).

Therefore, we chose the preoperative HOOS as the questionnaire with the best correlation to responder status and generated a multivariable analysis including different possible confounders. The results revealed preoperative HOOS as an independent parameter correlating with postoperative responder status (OR = 0.76, 95% CI = 0.66 – 0.88, p<0.001) whereas all other variables showed no association (Table 4).

**Figure 3:**
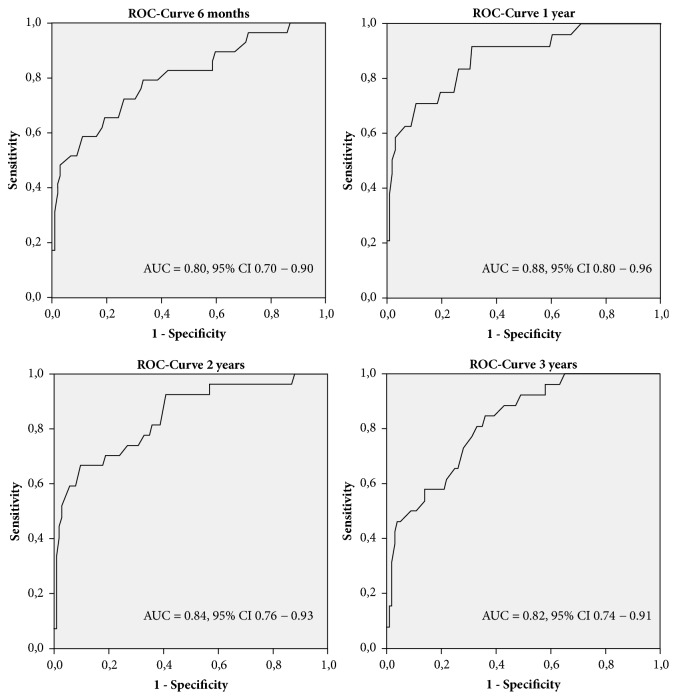
ROC-curve analysis for nonresponder at the different follow-up points using preoperative HOOS.

To define cutoff values for HOOS to predict postoperative outcome one year after surgery, a ROC-curve plot was generated (AUC = 0.88, 95% CI = 0.80 – 0.96). Analyzing one-year nonresponders, Youden Index was highest with 0.61 for a benchmark HOOS value of 40.3. This resulted in a sensitivity of 91.7% and specificity of 68.9% to identify nonresponders. Accordingly, the negative predictive value was 97.4% and the positive predictive value 39.7%. To test the validity of the cutoff, ROC-curve analysis for other follow-up points was performed (Figure 3). Nonresponders as defined 3 years after surgery were predictable with a sensitivity of 80.8% and a specificity of 67.0% using a cutoff for preoperative HOOS of 40.3.

## 4. Discussion

THR is a frequently performed procedure in orthopaedic surgery [2, 35]. Since clinical resources are limited [5], preoperative predictors of outcome for THR play an important role when counselling patients in the office [12]. In the current study, we aimed (1) to analyze different preoperative measures regarding their ability to predict outcome and (2) to define benchmarks for preoperative measures to identify patients preoperatively associated with high improvement after THA. We found the preoperative HOOS as the questionnaire with the highest predictive power among all other preoperative measures. A cutoff HOOS value of 40.3 resulted in a sensitivity of 91.7% and specificity of 67.9% to identify nonresponders after THA.

In answer to the first question of the study, which preoperative measure shows the best association to positive responder status after THA, we found a difference in preoperative scores (HOOS, EQ-5D, PCS) between responders and nonresponders as defined by the modified OMERACT-OARSI criteria after THA. Preoperative HOOS, EQ-5D, and PCS were consistently different between responders and nonresponders for all follow-up points. In contrast, preoperative mental health as measured in the MCS showed no association with responder status as defined at the different follow-up points. This is in line with previous studies revealing a correlation between high preoperative measures and worse clinical outcome after joint replacement [11, 12]. However, not all preoperative measures seem to have this predictive effect [15]. On the other hand patients with poor preoperative function have high expectations of THA [36]. This might result in unrealistic expectations and thus dissatisfaction after surgery. However, greater numbers of preoperative expectations were reported in literature to be associated with improvement after THA [37]. In contrast to our study results, a previous study described a correlation between preoperative MCS and responder status. However, a different cohort dependent definition for good outcome was used (MID) [11]. Another study found no correlation between preoperative MCS and postoperative responder grade. This study used the lowest quartile criteria [6]. The reason why there was no relation between preoperative MCS and responder status in our study might rely on the applied definition of responder. Since the definition is mainly based on parameters of physical function, psychological effects might not be accounted for appropriately.

Among the different preoperative measures preoperative HOOS showed the highest predictive power for positive responder as defined at all follow-up points as measured by logistic regression analysis. In literature parameters associated with outcome after THA such as gender, age, Kellgren Score, pain, or ASA class have been described. In the present study, the correlation of preoperative HOOS and responder status was independent of these potential confounders as demonstrated by multivariable analysis. Neither gender, age, Kellgren Score, pain nor ASA class correlated with responder status after THA. In contrast to our results, women and patients at an advanced age were previously described in literature to be associated with lower improvement in physical function [12, 38], whereas more severe radiographic degeneration preoperatively correlated with better functional outcome after THA in former studies [12, 38]. Patient comorbidity and number of troublesome joints also correlated with responder grade after total joint replacement [11]. Previous trauma [12] and higher preoperative pain [6] were reported as risk factors for worse outcome. However, due to the strict inclusion criteria of the present study, these parameters have not been addressed.

Researching into a benchmark to distinguish between responders and nonresponders, ROC-curve analysis showed a good discriminatory ability with an AUC of 0.88. For a cutoff of 40.3 in preoperative HOOS, the sensitivity was 91.7% and the specificity 68.9%. Therefore, patients with a lower preoperative HOOS value than 40.3 have a high probability for excellent improvement in pain and function if undergoing THA. This should be considered when counselling patients in the office since the HOOS is easy to obtain in the preoperative situation. Comparing a different predictive model stated in literature, there was an AUC of 0.76 with a sensitivity of 66.1% and specificity of 74.3% [12]. SF-36 physical function score, sex, age, radiographic grade, previous hip injury, and number of painful joints were included in this clinical risk scoring tool [12]. In a different study, gait analysis was used to predict clinical response after THA. In combination with preoperative HHS, nonresponders were identified with a sensitivity of 71.4% and specificity of 99.1% [32]. Due to our definition of positive responder comprising a high improvement in both pain and function, we aimed to create a model with a high negative predictive value. The negative predictive value in our study was 97.4%. This means a patient with a preoperative HOOS below 40.3 has a probability of 97.4% for a positive response after THA. This could facilitate handling medical indication of THA for the orthopaedic surgeon when counselling patients in the office. The low positive predictive value of 39.7% shows that a HOOS value above 40.3 does not necessarily mean a patient will not become a responder after surgery. However, this was not the intention of our study since we focused on an easily applicable tool to identify patients benefitting best from THA.

There are several limitations of this study. First, the results depend on the applied definition of responder status after THA. To minimize potential bias, we chose patient characteristics independent dichotomization for responders in contrast to cohort dependent dichotomization such as lowest quartile or minimal important difference (MID). Using non-cohort dependent benchmarks should maximize generalizability [32]. According to the applied definition of responder grade which is mainly based on physical function, the results might be susceptible to potential bias. Second, due to the low numbers of nonresponders according to the original OMERACT-OARSI criteria [26], we set the requirements stricter. Therefore according to this definition, a positive responder status means patients with high improvement in both function and pain after THA. Third, the current analysis is restricted to the information provided by the data collected during the course of the study. More detailed information on the patient's psychological or social status might have an impact on the patient specific outcome and improve prediction of outcome. Fourth, for the current analysis only mid-term outcome data for the first 3 years are available. It would have been of interest to include long-term outcome and failure rates. Fifth, due to the strict inclusion and exclusion criteria of the main study, the study population represents a highly selected patient subgroup. Therefore, the results cannot be automatically transferred to each individual patient undergoing THR. A strength of the study is the fact that all data refer to one single university medical center reflecting a specific operative workflow for THA as well as an identical postoperative treatment protocol for all patients. Similarly, components of a single manufacturer were used. All this contributes to minimizing confounding factors.

## 5. Conclusions

In conclusion, presurgical HOOS can predict outcome better than other preoperative outcome measures in this selected group of patients undergoing THA. According to the applied definition of responder, the preoperative HOOS showed the best predictive power. Patients with a preoperative HOOS above 40.3 could be identified as nonresponders with a sensitivity of 91.7% and a specificity of 68.9%. Therefore, preoperative HOOS should be considered when counselling patients in the office. Further studies are required to reveal the generalizability of the study results.

## Figures and Tables

**Table 1:** Anthropometric and operative characteristics of the study group classified in responder grade one year after THA*∗*.

N=126	Responders	Non-Responders^#^
Age (years)	61.6 ± 7.4	64.2 ± 8.2
Gender (men/women)	51/52	12/11
BMI (kg/m2)	27.0 ± 4.1	26.6 ± 3.5
Treatment side (left/right)	48/55	11/12
ASA Class 1	18 (17.5%)	7 (30.4%)
ASA Class 2	53 (51.5%)	12 (52.2%)
ASA Class 3	32 (31.1%)	4 (17.4%)
Kellgren–Lawrence score	9 (5–10)	8 (7–9)
Length of skin incision (cm)	10.4 ± 1.2	10.2 ± 1.4
Operative time (minutes)	68.7 ± 14.7	66.3 ± 13.7

*∗* For categorical data, values are given as relative and absolute frequencies; for quantitative data, values are given as mean (standard deviation) or median (range). # Responders and nonresponders were defined according to the modified OMERACT-OARSI criteria. THA: total hip arthroplasty, BMI: body mass index, ASA: American Society of Anaesthesiologists.

**Table 2:** Comparison of presurgical measures in relation to responder status defined separately for each follow-up point *∗*.

**Responder Status**			**HOOS**	**EQ-5D**	**PCS**	**MCS**
**6 months** n=128	Non-Responder	median	49.4	0.79	33.1	38.0
		range	22.5	0.11	16.0	-26.0
			77.5	0.89	49.0	82.0
	Responder	median	35.0	0.70	26.5	38.0
		range	1.9	0.11	14.0	-40.0
			58.8	1.00	44.0	82.0
		p	<0.001	0.06	<0.001	0.46

**1 year** n=126	Non-Responder	median	52.9	0.79	34.4	48.0
		range	30.0	0.18	24.0	-6.0
			77.5	0.89	49.0	82.0
	Responder	median	35.0	0.70	26.7	38.0
		range	1.9	0.11	14.0	-40.0
			58.8	1.00	44.0	82.0
		p	<0.001	0.01	<0.001	0.96

**2 years** n=126	Non-Responder	median	50.0	0.79	32.7	48.0
		range	22.5	0.11	16.0	-26.0
			77.5	0.89	49.0	82.0
	Responder	median	35.0	0.70	26.8	38.0
		range	1.9	0.11	14.0	-40.0
			64.4	1.00	44.0	82.0
		p	<0.001	0.01	0.001	0.73

**3-years** n=125	Non-Responder	median	48.8	0.79	32.9	53.0
		range	31.3	0.18	16.0	3.0
			77.5	0.89	49.0	82.0
	Responder	median	35.0	0.70	26.5	38.0
		range	1.9	0.11	14.0	-40.0
			64.4	1.00	44.0	82.0
		p	<0.001	0.002	<0.001	0.36

*∗* For quantitative data, values are given as median and range. HOOS: Hip Disability and Osteoarthritis Outcome Score, EQ-5D: EuroQol, PCS: Physical Component Summary of the Short Form 36 questionnaire, MCS: Mental Component Summary of the Short Form 36 questionnaire, p: p-value.

**Table 3:** Binary logistic regression for each presurgical measure for responder at each follow-up after THA.

	**R-Squared**	**Effect Size**	**OR**	**95**%** CI**		**P-value**
	**6 months**					

**HOOS**	0.34	0.72	0.89	0.84	0.93	<0.001
**EQ-5D**°	0.05	0.23	0.99	0.97	1.00	0.06
**PCS**	0.19	0.48	0.89	0.83	0.95	<0.001

	**1 year**					

**HOOS**	0.48	0.96	0.84	0.78	0.90	<0.001
**EQ-5D**°	0.09	0.31	0.98	0.96	1.00	0.01
**PCS**	0.27	0.61	0.86	0.79	0.92	<0.001

	**2 years**					

**HOOS**	0.40	0.82	0.87	0.82	0.92	<0.001
**EQ-5D**°	0.08	0.29	0.98	0.96	1.00	0.02
**PCS**	0.16	0.44	0.90	0.84	0.96	0.001

	**3 years**					

**HOOS**	0.35	0.73	0.88	0.83	0.93	<0.001
**EQ-5D**°	0.11	0.35	0.97	0.95	0.99	0.01
**PCS**	0.17	0.45	0.89	0.84	0.95	0.001

THA: total hip arthroplasty, OR: odds ratio, CI: confidence interval, HOOS: Hip Disability and Osteoarthritis Outcome Score, EQ-5D°: EuroQol multiplied by 100, PCS: Physical Component Summary of the Short Form 36 questionnaire.

**Table 4:** Multivariable analysis of risk factors associated with responder grade one year after THA.

**Responder**	**OR**	**95**%** CI**		**P-value**
HOOS	0.76	0.66	0.88	<0.001
Gender	0.87	0.20	3.81	0.86
Age	0.92	0.83	1.03	0.13
ASA	1.18	0.37	3.75	0.78
BMI	0.85	0.67	1.07	0.17
Operative time	1.01	0.95	1.08	0.77
Kellgren–Lawrence score	1.82	0.72	4.64	0.21
Length of skin incision	0.84	0.43	1.65	0.61
THR	0.99	0.94	1.04	0.71
VAS	5.56	0.03	1220.58	0.53
MCS	1.04	0.98	1.11	0.20

THA: total hip arthroplasty, OR: odds ratio, CI: Confidence Interval, HOOS: Hip Disability and Osteoarthritis Outcome Score, ASA: American Society of Anaesthesiologists, BMI: body mass index, THR: Total Hip Replacement Expectations Survey, VAS: visual analogue scale, MCS: Mental Component Summary of the Short Form 36 questionnaire.

## Data Availability

The data used to support the findings of this study are available from the corresponding author upon request.
